# The Effectiveness and Cost-Effectiveness of Hepatitis C Screening for Migrants in the EU/EEA: A Systematic Review

**DOI:** 10.3390/ijerph15092013

**Published:** 2018-09-14

**Authors:** Christina Greenaway, Iuliia Makarenko, Claire Nour Abou Chakra, Balqis Alabdulkarim, Robin Christensen, Adam Palayew, Anh Tran, Lukas Staub, Manish Pareek, Joerg J. Meerpohl, Teymur Noori, Irene Veldhuijzen, Kevin Pottie, Francesco Castelli, Rachael L. Morton

**Affiliations:** 1 Division of Infectious Diseases, Jewish General Hospital, McGill University, Montreal, QC H3T 1E2, Canada; 2 Centre for Clinical Epidemiology of the Lady Davis Institute for Medical Research, Jewish General Hospital, Montreal, QC H3T 1E2, Canada; makarenko.j@gmail.com (I.M.); Balqis.alabdulkarim@mail.mcgill.ca (B.A.); 3 Department of Epidemiology, Biostatistics, and Occupational Health, McGill University, Montreal, QC H3A 1A2, Canada; apalayew@gmail.com; 4 Department of Microbiology and Infectious Diseases, Université de Sherbrooke, Sherbrooke, QC J1H 5N4, Canada; Claire.Nour.Abou.Chakra@USherbrooke.ca; 5 Musculoskeletal Statistics Unit, The Parker Institute, Bispebjerg and Frederiksberg Hospital & Department of Rheumatology, Odense University Hospital, DK2000 Odense, Denmark; Robin.Christensen@regionh.dk; 6 NHMRC Clinical Trials Centre, The University of Sydney, Sydney 1450, Australia; anh.tran@ctc.usyd.edu.au (A.T.); lukas.staub@ctc.usyd.edu.au (L.S.); Rachael.morton@ctc.usyd.edu.au (R.L.M.); 7 Department of Infection, Immunity and Inflammation, University of Leicester, Leicester LE1 7RH, UK; manish.pareek@leicester.ac.uk; 8 Institute for Evidence in Medicine (for Cochrane Germany Foundation), Medical Center, University of Freiburg, 79110 Freiburg, Germany; meerpohl@ifem.uni-freiburg.de; 9 European Centre for Disease Prevention and Control, 169 73 Solna, Sweden; teymur.noori@ecdc.europa.eu; 10 Centre for Infectious Disease Control, National Institute for Public Health and the Environment (RIVM), 3720 BA Bilthoven, The Netherlands; irene.veldhuijzen@rivm.nl; 11 C.T. Lamont Primary Health Care Research Centre, Bruyère Research Institute, Ottawa, ON K1N 5C8, Canada; kpottie@uottawa.ca; 12 Centre for Global Health, University of Ottawa, Ottawa, ON K1N 5C8, Canada; 13 Division of Infectious Diseases, University of Brescia, 255123 Brescia, Italy; francesco.castelli@unibs.it

**Keywords:** hepatitis C, screening, migrants, viral hepatitis elimination, European Union

## Abstract

Chronic hepatitis C (HCV) is a public health priority in the European Union/European Economic Area (EU/EEA) and is a leading cause of chronic liver disease and liver cancer. Migrants account for a disproportionate number of HCV cases in the EU/EEA (mean 14% of cases and >50% of cases in some countries). We conducted two systematic reviews (SR) to estimate the effectiveness and cost-effectiveness of HCV screening for migrants living in the EU/EEA. We found that screening tests for HCV are highly sensitive and specific. Clinical trials report direct acting antiviral (DAA) therapies are well-tolerated in a wide range of populations and cure almost all cases (>95%) and lead to an 85% lower risk of developing hepatocellular carcinoma and an 80% lower risk of all-cause mortality. At 2015 costs, DAA based regimens were only moderately cost-effective and as a result less than 30% of people with HCV had been screened and less 5% of all HCV cases had been treated in the EU/EEA in 2015. Migrants face additional barriers in linkage to care and treatment due to several patient, practitioner, and health system barriers. Although decreasing HCV costs have made treatment more accessible in the EU/EEA, HCV elimination will only be possible in the region if health systems include and treat migrants for HCV.

## 1. Introduction

Chronic hepatitis C is an important public health problem in the EU/EEA, with an estimated 3.24 million persons having active hepatitis C virus (HCV) infection [1,2]. It is a leading cause of chronic liver disease and liver cancer in the EU/EEA due to undetected and untreated infections [3,4,5]. Since 2013, the landscape of HCV treatment has changed rapidly as pan-genotypic DAA HCV treatment regimens that cure most infections (>95% of cases) have become available, making HCV elimination possible [6,7,8,9]. In 2015 however, only 34% of HCV infected persons had been diagnosed and less than 5% of all HCV cases had been treated [2]. Identifying and treating all groups at risk for HCV in the EU/EEA will be essential to address the health and economic burden due to HCV in the EU/EEA and to reach WHO elimination goals by 2030 [3,5,9,10,11].

HCV screening and control programs in the EU/EEA primarily focus on persons who inject drugs (PWID), as they are the largest and highest burden population [1]. Migrants from intermediate and high HCV prevalence countries (anti-HCV ≥ 2% and ≥5%, respectively) are an additional important and underappreciated group at increased HCV risk in the EU/EEA and often do not have identifiable HCV risk factors [12,13]. They are most likely to have been exposed to HCV in their countries of origin through receipt of contaminated blood products or unsafe injections or procedures, and have a prevalence of HCV that reflects that of their countries of origin [14,15]. The increased flow of migrants from intermediate and high HCV prevalence countries into the EU/EEA over the past few decades has resulted in a disproportionately high number of reported HCV cases (14%) occurring among migrants, who account for up to one half of all cases in some low HCV prevalence EU/EEA countries [12,13]. HCV diagnosis among migrants living in low incidence countries is delayed due to several patient, practitioner, and infrastructural barriers that may result in a higher burden of liver-associated complications compared to host populations [16,17]. We conducted a systematic review (SR) to estimate the effectiveness, resource use, costs, and cost-effectiveness of HCV screening programs for migrants in the EU/EEA.

## 2. Methods

### 2.1. Overall Approach and Key Questions

Using the Grading of Recommendations Assessment, Development, and Evaluation (GRADE) approach, the Campbell and Cochrane Collaboration Equity Methods Group and review team including clinicians, public health experts and researchers from across the EU/EEA, we conducted evidence syntheses. “Migrants”, a focus of this review, included asylum seekers, refugees, undocumented migrants, and other foreign-born residents. A detailed description of the methods have been published and were registered in PROSPERO (CRD42016045798) [18]. 

We used the GRADE approach to rate the certainty of evidence starting with a simplified categorization of study types (i.e., meta-analyses, RCTs, and observational studies). The rating scheme allows for factors that may raise or lower the level of certainty. Factors that lower certainty of evidence include, risk of bias, inconsistency across studies, indirectness, and publication bias. Factors that increase certainty of evidence include large effect size and an observed dose-response effect. The final certainty ratings are reflective of the certainty in the estimated effect in the context of bias and limitations. Evidence was graded as high, moderate, low, or very low certainty, based on how likely further research would change the confidence in the estimate of effect. Low certainty and very low certainty do not mean absence of evidence for effectiveness, but rather signal highlights the need for more research to improve the precision of the estimate of effect.

This review followed the Grading of Recommendations Assessment, Development, and Evaluation (GRADE) and Cochrane methodological approach [18]. We used the Preferred Reporting Items for Systematic Reviews and Meta-Analyses (PRISMA) Checklist for reporting the results of the systematic reviews (SR) [18,19]. The review team developed two overarching research questions (PICO: Population, Intervention, Comparison, Outcome) and a logic model (Figure S1). The logic model showed key questions/concepts along the evidence chain along the screening effectiveness pathway [18,20,21]. The two overarching research questions (PICO) we sought to answer were:

- What is the effectiveness of screening migrants arriving and living in the EU/EEA for HCV?
- What is the cost, resource utilization, and cost-effectiveness for screening migrants for HCV?

The following key questions were identified along the screening effectiveness pathway. (1) What is the test accuracy and performance characteristics of screening tests for HCV? (2) What is the efficacy of new direct acting antiviral (DAA) treatments for HCV to decrease HCV associated morbidity and mortality? (3) What is the uptake of HCV of screening and treatment? (4) What is the cost-effectiveness of a screen-treat approach for HCV in the general population and the migrant population when treated with DAAs? [18].

### 2.2. Search Strategy and Selection Criteria

We conducted two searches, one for SRs and guidelines on the effectiveness and cost-effectiveness of HCV screening programs in migrants and a second search for SRs and primary studies on the resource use, costs, and cost-effectiveness of HCV screening programs in migrants. For the first search, Medline via OVID, EMBASE, CINAHL, Epistemonikos, and Cochrane CENTRAL were searched for publications between 1 January 2010 and 12 May 2016. A combination of key terms was used including “hepatitis C/HCV”, “screening”, “migrants”, “costs”, “cost-effectiveness” AND “guidelines”, and “reviews”. The search terms and the search strategy for Ovid Medline are included in the supplementary material (Table S1). We also searched grey literature websites for published guidelines and reports at CDC, ECDC, EASL (European Association for the Study of the Liver), and WHO. We applied no language restrictions to the search. In the second search, using terms of “hepatitis C/HCV”, “screening”, “costs”, and “cost-effectiveness”, Medline via Ovid, EMBASE, the NHS Economic Evaluation Database (NHS EED), Database of Abstracts of Reviews of Effects (DARE), and the Cost Effectiveness Analysis Tufts registry and Google scholar databases were searched for publications between 1 January 2000 and 31 May 2016. Reference lists of relevant reviews were also searched.

### 2.3. Study Selection and Quality Assessment

**Figure 1:**
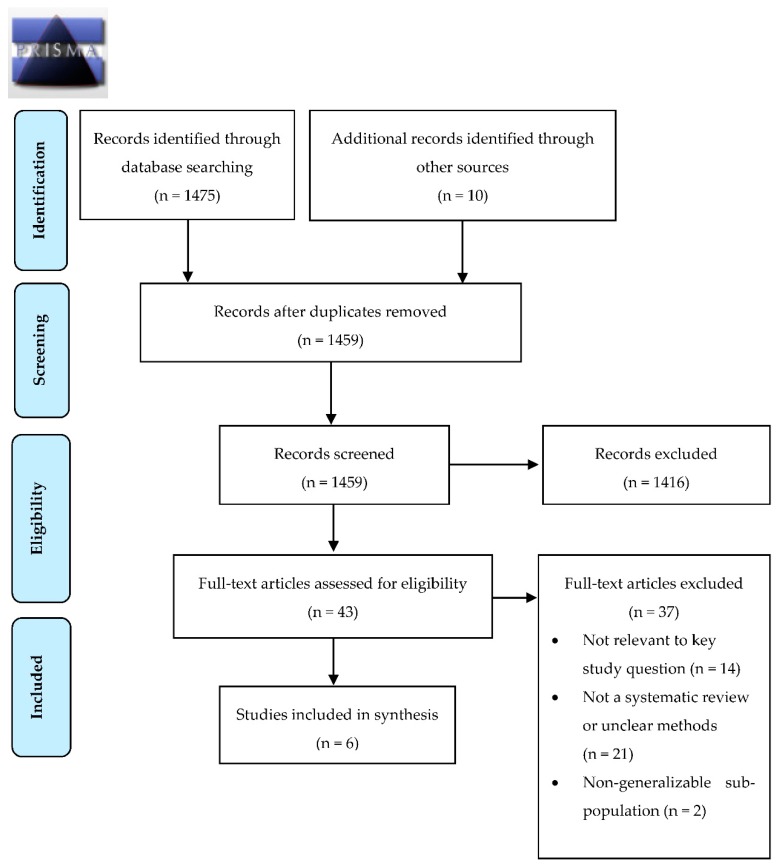
Preferred Reporting Items for Systematic Reviews and Meta-Analyses (PRISMA) diagram for the effectiveness of hepatitis C screening.

**Figure 2:**
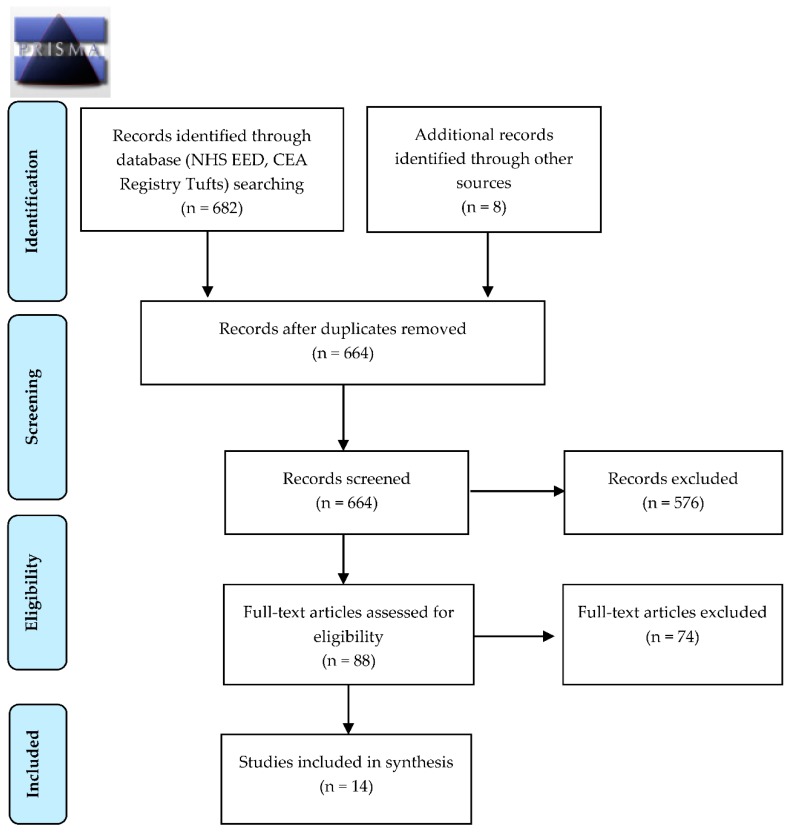
PRISMA diagram for the resource use, costs, and cost-effectiveness for hepatitis C screening.

Two authors screened the titles and abstracts, assessed selected full-text articles for eligibility, and extracted data from included articles. Disagreements were resolved by consensus or by a third author. For the screening effectiveness search we included systematic reviews on the impact of HCV screening or antiviral therapies on the development of liver related morbidities such as cirrhosis, hepatocellular carcinoma, and the need for liver transplantation and all-cause or attributable mortality. For the cost-effectiveness search we included individual economic studies of screening strategies that included an arm of direct acting antiviral (DAA) therapies or studies of the cost-effectiveness of DAA therapies [8]. We only included studies published in full and in English or French. If more than one version of a SR was identified, the most recent was considered. Studies were excluded if they focused only on nongeneralizable subgroups (such as PWIDs) (Figure 1 and Figure 2).

The methodologic quality of SRs was assessed by two authors using the AMSTAR tool (A Measurement Tool to Assess Systematic Reviews) [22]. The GRADE criteria were applied to assess the certainty of evidence in preselected outcome measures in the SRs (GRADE Tables S2–S5) [23]. For the second search which included individual studies the certainty of economic evidence in each study was assessed using the relevant items from the 1997 Drummond checklist [24].

### 2.4. Data Extraction and Synthesis

The following information was extracted from each study: study design, objectives, analyses, quality of the individual studies included in the systematic review, population examined, number of included studies, total number of participants included, intervention, outcome, and the results. For the economic studies we extracted the following data; economic methods used (e.g., microcosting study, within-trial cost–utility analysis, Markov model), description of the case base population, the intervention and comparator, absolute size and relative difference in resource use, cost-effectiveness results (e.g., incremental net benefit (INB) or incremental cost-effectiveness ratio (ICER)), and three specific questions for the GRADE Evidence to Decision table: the size of the resource requirements, the certainty of evidence around resource requirements, and whether the cost-effectiveness results favored the intervention or comparison [25]. Key results were converted to 2015 Euros using the Cochrane web-based currency conversion tool: https://eppi.ioe.ac.uk/costconversion/default.aspx.

## 3. Results

### 3.1. Search Results

In the search for the effectiveness of HCV screening we retrieved 1475 references and identified 10 additional records through other sources (Figure 1). After duplicates were removed, 1459 references were screened by title and abstract. A total of 43 references were then selected for full text assessment. We did not identify any randomized controlled trials or SRs on the effectiveness of HCV screening in the general or migrant populations. We therefore included five SRs and one guideline that addressed the key questions along the screening evidence chain: the performance of HCV diagnostic tests (n = 2) [6,26], the impact of HCV treatment on preventing HCC and all-cause mortality (n = 3) [27,28,29], and the HCV care continuum (n = 1) (Table 1) [30]. In the economic search, 682 articles were retrieved and an additional eight records identified through other sources (Figure 2). After duplicate removal, 664 references were screened by title and abstracts. Of these, a total of 88 references underwent full text assessment and 14 individual studies of populations living in low HCV prevalence countries were included (Table 2) [31,32,33,34,35,36,37,38,39,40,41,42,43,44].

### 3.2. Performance of Diagnostic Tests

The performance of diagnostic testing for HCV has been recently summarized in the 2017 WHO Guidelines on Hepatitis B and C testing [6]. WHO estimates the sensitivity and specificity of 3rd generation HCV enzyme immunoassays (EIA) to be 98% and 99%, respectively [6]. Similarly, the sensitivity of the confirmatory test to detect virus nucleic acid (nucleic acid test; NATs) is estimated to be 96.2% (95% CI, 94.4–97.5) and the specificity to be 98.9% (98.3–99.3) [6]. There was no reported evidence suggesting that migrants or any other group would encounter lower performance rates. The accuracy of point of care testing, a strategy that potentially could increase screening uptake, was reviewed by Khuroo et al. who found that these tests performed well in low, middle, and high income countries [26]. The sensitivity (95.8% (93.9–97.1)) was slightly lower compared to EIAs but demonstrated comparable specificity (99.0% (98.5–99.3)) [26]. The GRADE certainty of the evidence in the Khuroo study was very low (S3 GRADE Table 1).

### 3.3. Impact of Therapy on Long-Term Outcomes

Interferon free DAAs are the recommended therapy for all HCV genotypes in the EU/EEA [8]. These regimens are well tolerated and cure > 95% of cases based on achieving sustained virologic response (SVR0, which is considered to be a reliable surrogate outcome for HCV cure) [7,8]. Within the search dates we did not identify any studies that examined the impact of DAA therapies on long-term HCV liver related outcomes or mortality, as these agents only became available in 2013. Three SRs that assessed the impact of older interferon (IFN) based HCV treatment on preventing liver related sequelae and all-cause mortality were included [27,28,29]. These three SRs included studies from Europe, North America, Australia, and Asia, and found that IFN-based HCV therapy significantly decreased rates of hepatocellular carcinoma (HCC), hepatic decompensation, and all-cause mortality in those on HCV treatment, and particularly in those who achieved SVR [27,28,29]. In a meta-analysis by Kimer et al., the risk of HCC was lower in those on antiviral HCV therapy (IFN or PEG-IFN alone or with ribavirin) compared to placebo or no intervention (RR = 0.53, 95% CI 0.34–0.81). This effect was much more pronounced among virological responders compared to nonresponders [27]. In the SR by Simmons et al., the adjusted hazard ratio (aHR) of all-cause mortality rate was lower in patients on treatment for chronic HCV after a median follow-up time of 5.4 years [28]. In those achieving SVR, compared with non-SVR, the aHR was 0.50 (95% CI: 0.37–0.67) in the general population, and 0.26 (95% CI: 0.18–0.37) in the cirrhotic group [28]. Finally, in the SR conducted by the Public Health Agency of Canada (PHAC), a reduction in hepatic mortality (60 fewer/1000, 95% CI: 59–62), HCC (18 fewer/1000, 95% CI: 17–19), hepatic decompensation or decompensated cirrhosis (46 fewer/1000, 95% CI: 46–47), and need for liver transplantation (4 fewer/1000, 95% CI: 4–5) among those treated with DAA to PEG-IFN was found [29]. The GRADE certainty of the evidence of the data in these three systematic reviews was very low to moderate (GRADE Tables S3–S5). 

### 3.4. The HCV Care Continuum and Pathway

In a SR of studies of the HCV care continuum in the pre-DAA period among in the general population in the US from 2003–2013, only 50% of HCV cases were diagnosed and aware of their infection, 27% had HCV RNA confirmatory testing, 16% were prescribed HCV therapy, and 9% achieved SVR [30]. The results of this study were not stratified by risk group nor did they report on the barriers to uptake of the steps along the care cascade. A modeling study in Europe published after the search timeframe also demonstrated a weak HCV care continuum in 2015, consistent with the US SR by Yehia [2]. They found that in Europe in 2015, only 36% of HCV infected persons have been screened and, of those diagnosed, only 12.7% and 11.3% had been treated and cured, respectively [2]. They found that, to achieve WHO elimination targets, expansion of screening programs in the EU/EAA would be needed along with unrestricted access to treatment for all found to be infected.

### 3.5. Resource Use, Costs and Cost-Effectiveness

Screening for HCV in those treated with DAAs is cost-effective even at higher 2015 costs. A UK study evaluated the cost-effectiveness of screening and treating pregnant women attending antenatal clinics [44]. The incremental cost effectiveness ratio (ICER) for screening and treatment (PR) compared with no screening and no treatment was £2400 (€2745) per QALY gained. For screening and treating with DAAs compared with no screening and no treatment, the ICER was still cost-effective at £9139 (€10,455)/QALY gained. A Canadian study evaluated the cost-effectiveness of screening for HCV in different age groups and then treating with DAAs in the Canadian population where the HCV seroprevalence ranged from 0.3 to 0.8% [32]. The ICER for IFN-free DAAs vs. older therapies ranged from CAN$34,359 (€21,977) to CAN$44,034 (€28,166) per QALY gained [32]. In the US, Rein et al. found that screening followed by DAA therapy was moderately cost-effective with ICERs ranging from US$47,237 (€40,665) to US$72,169 (€62,128) per QALY gained [36].

The Drummond Criteria [24]: (i) Was a well-defined question posed in answerable form? (ii) Was a comprehensive description of the competing alternatives given (i.e., can you tell who did what to whom, where, and how often)? (iii) Was the effectiveness of the program or services established? (iv) Were all the important and relevant costs and consequences for each alternative identified? (v) Were costs and consequences measured accurately in appropriate physical units (e.g., hours of nursing time, number of physician visits, lost working days, and gained life years)? (vi) Were the cost and consequences valued credibly? (vii) Were costs and consequences adjusted for differential timing? (viii) Was an incremental analysis of costs and consequences of alternatives performed? (ix) Was allowance made for uncertainty in the estimates of costs and consequences? (x) Did the presentation and discussion of study results include all issues of concern to users?

Non-pan-genotypic DAA therapies have also been found to be moderately cost-effective, however they had a large budget impact at the 2015 cost of treatment [31]. Deuffic-Burban found that DAAs were moderately cost-effective for genotypes 1 and 4 regardless of fibrosis stage, ranging from €40,000 to €88,000 per quality-adjusted life year (QALY) gained (Table 2). IFN-based regimens were estimated to be more cost-effective for genotypes 2 or 3 at €21,300 to €19,400 per QALY gained regardless of fibrosis stage [31]. DAAs were found to be moderately cost-effective for genotype 1 & 4 at a median threshold of €24,000/QALY gained and maximum upper limit of €80,000/QALY gained however, introducing these regimens on a wide scale would have a substantial budget impact of €3.5–7.2 billion on the French health care system [31,45]. Several US studies have evaluated the cost-effectiveness of DAA therapies compared to older PEG-INF-RBV therapies and found that DAA therapies were moderately cost-effective at a willingness-to-pay threshold of $50,000 US (€39,210), but varied significantly by HCV genotype, presence of liver fibrosis, and treatment history [33,34,35,36,37,38]. In the US, DAAs were moderately cost-effective for genotypes 1 & 4 whereas IFN-RBV was more cost-effective for genotypes 2 & 3. DAAs were more cost-effective in the presence of cirrhosis and treatment naïve patients. Providing DAAs to all eligible HCV patients would also have a huge budget impact, costing an additional US$65 (€56) billion over a 5-year period, whereas the resulting cost-offsets were estimated at only US$16 (€14) billion [34].

## 4. Discussion

The data in this review supports the effectiveness and cost-effectiveness of HCV screening in populations at risk for HCV infection, including migrants from intermediate and high HCV prevalence countries (anti-HCV ≥ 2% and ≥5%, respectively). Screening tests to detect the presence of HCV antibodies performed equally well in all populations. In the laboratory setting or at the point-of-care both are highly sensitive and specific [6,26]. DAA therapies are highly efficacious and well tolerated with >95% cure rates in a range of populations in different countries [7]. Achieving SVR with HCV therapy is associated with decreased risk and rate of liver disease progression, lower rates of HCC development, and improved survival [27,28,29,46]. At 2015 costs, DAA based regimens were only moderately cost-effective and as a result less than 30% of those with HCV had been screened and less than 5% of all HCV cases had been treated in the EU/EEA in 2015 [2,31]. Migrant populations in the EU/EEA face difficulties accessing care and treatment as a result of numerous barriers at the patient, provider, and health system level [16,47]. To reach HCV elimination goals in the EU/EEA dramatic scale up of HCV testing with diagnosis of all groups at HCV risk, including migrants, and linking those found to be positive to care and treatment will be required.

Migrants living in the EU/EEA bear a disproportionate burden of HCV [1,13]. They are older and more likely to have advanced liver disease and hepatocellular carcinoma compared to non-migrants at the time of HCV diagnosis [17,48,49]. This is likely due to missed or delayed diagnoses. In a survey in Finland, 63% of migrants found to be HCV positive had not been previously diagnosed [50]. Seventy percent of these HCV positive migrants had been living in Finland for more than five years. Similarly, in a population based study in Canada, it took a mean of 10 years after arrival for migrants to be diagnosed with HCV [48]. Another Canadian study found that it was cost-effective to screen immigrants for HCV followed by DAA treatment at an anti-HCV prevalence of 1.9%, which is the mean HCV seroprevalence of migrants living in the EU/EEA [12,13,51]. These data taken together suggest that early screening of migrants based on the HCV prevalence in the country of origin with linkage to care and treatment could prevent liver related sequelae in the migrant population and would be cost-effective. In an ECDC survey, 18 of 21 responding countries had national guidance on HCV testing; however, only six countries (29%) had guidance on testing migrants for HCV [52]. This highlights an important gap and an opportunity for health promotion among the migrant population. 

Migrants face multiple barriers in accessing healthcare services resulting in gaps along all steps of the HCV care continuum [16,53]. Many groups of newly arriving migrants, including asylum seekers and undocumented migrants, lack entitlement to health care in the EU/EEA, thus preventing them from being diagnosed or receiving treatment [54]. Individual barriers include lack of knowledge and awareness of risk factors, fear, and stigma of blood-borne diseases, and socioeconomic, linguistic, and cultural barriers [47,55,56,57]. Providers are frequently unaware that birth in an HCV endemic country is an important risk factor for HCV. Language and cultural discordance between patients and providers may lead to poor communication and low quality of care [16,53,58]. Lack of screening guidelines and programs and the lack of access to interpreters are important health system barriers. Barriers to effective screening and treatment programs may also include lack of political will to address migrant health issues or negative national attitudes toward migrants. Resulting policies may deny migrants the entitlement to health care or prevent the development of migrant-friendly heath care systems [58].

Evidence from primary studies of migrant populations suggests that HCV screening uptake and linkage to care could be improved by implementing decentralized community-based screening strategies, and cooperation with community-based organizations to overcome cultural and language barriers [59,60,61,62,63,64]. Furthermore, integrated point-of-care testing for HCV, HBV, and HIV increased testing uptake [59,65]. HCV screening and treatment programs for migrants in the EU/EEA will need to be tailored to their specific needs. In addition, it will be necessary to ensure universal access to health care in order to enhance uptake along the entire HCV care continuum.

The greatest barrier to scaling up HCV treatment in the EU/EEA has been the high cost of therapy, which has decreased dramatically in the past two years [66]. As a result, many countries in the EU/EEA have lifted restrictions and provide more widespread availability of HCV therapy in the region [67]. Dropping prices and simpler short treatment regimens have helped create an opportunity to eliminate HCV in the EU/EEA. Recent guidance from the WHO has highlighted the need to increase HCV case detection and linkage to HCV care [6]. This includes screening persons originating from countries with an intermediate (≥2%) and high (≥5%) HCV prevalence, which includes many of the migrants living in the EU/EEA. Consideration of existing prevention and control efforts and the capacity of existing systems must also be taken into account [68]. Knowledge of the HCV epidemiology in each EU/EEA Member State will be needed to identify those migrant groups at highest HCV risk, given that the top countries of origin of HCV infected migrants in each country varies (Table S6) [13]. Each country will need to assess their own capacity to increase HCV testing in at risk populations and to ensure that programs are in place that effectively link those with active HCV to care and provide HCV treatment.

### Strengths and Limitations

The major strength of our study is that we used a systematic review process to identify relevant studies and the GRADE methodology to evaluate the certainty of the evidence. Our study had the following limitations. The findings were limited by the low to moderate certainty of the evidence of the included studies. Our study was also limited by the low number of studies reporting on linkage to care for migrants to EU/EEA. Finally, the included cost-effective studies modeled the 2015 cost of DDA therapies. We anticipate however, that HCV screening and treatment will be even more cost-effective given the dramatic decrease in cost of DAA therapy in the EU/EEA since 2015 [66,67].

## 5. Conclusions

In many EU/EEA countries migrants originating from intermediate and high HCV prevalence countries make up a large proportion of all HCV cases and have poorer liver related outcomes due to delayed diagnosis and treatment. This health disparity is due to the numerous barriers migrants face accessing HCV diagnosis, care, and treatment. Migrant focused programs will need to ensure entitlement to health services and will be most effective if they address linguistic and cultural barriers, are community-based, and integrated with screening for other diseases such as HIV and HBV. Although decreasing HCV costs have made treatment more accessible in the EU/EEA, HCV elimination will only be possible in the region if health systems include and treat migrants for HCV [66].

## Figures and Tables

**Table 1:** Characteristics of studies on the effectiveness of hepatitis C screening.

Study	Quality of Systematic Review/GRADE Certainty of Evidence	Design	Population	Intervention/Outcomes	Results
Khuroo 2015 [26]	**Quality of systematic Review** AMSTAR: 8/11 **GRADE Certainty of Evidence** Very Low	Systematic Review Up to March 2012. N = 30 studies: 25—full-text article 2—WHO reports 1—WHO draft report 2—letters to editor	Adults > 18 years 17,151 participants 16 studies were conducted in low/middle income countries (India n = 4, Brazil n = 2, Cameroon n = 2, China n = 2, Egypt n = 1, Malawi n = 1, South Korea n = 2, Thailand n = 1, and Zimbabwe n = 1) 14 studies conducted in high income countries (United States n = 8, Germany n = 1, Italy n = 1, Spain n = 1) (Report for WHO n = 3)	**Intervention:** Point-of-care test: any commercially available assay at or near the site of patient care with <30 min turn-around time. Reference standard: third generation EIA, microenzyme immunoassay, CIA, RIBA, NAT **Outcome:** Sensitivity, specificity, LR+/−, Diagnostic OR (95% CI)	Pooled sensitivity: 97.4% (95% CI = 95.9–98.4) specificity: 99.5% (99.2–99.7) +LR: 80.17 (55.35–116.14) −LR: 0.03 (0.02–0.04) Diagnostic OR: 3032.85 (1595.86–5763.78) OraQuick test had the highest sensitivity and specificity: sensitivity: 99.5 (98.9–99.8) specificity: 99.8 (99.6–99.9)
Kimer 2012 [27]	**Quality of systematic Review** AMSTAR: 7/11 **GRADE Certainty of Evidence** Very Low	Systematic Review Up to 2012 N = eight RCTs, five prospective cohorts	RCTs conducted in France, Italy, Spain, Japan, and USA in patients with HCV-related cirrhosis or fibrosis and treated with antiviral therapy 1156 patients with therapy 1074 controls Prospective cohorts: Patients with HCV-related cirrhosis	**Intervention:** Antiviral therapy (PR, IFN, PEG-IFN) **Outcome:** RR, 95% CI of HCC development; number needed to treat to prevent 1 case of HCC = 1/risk difference overall mortality liver-related mortality liver-related morbidity	**Risk of HCC among received antiviral therapy vs. did not receive:** Absolute number of HCC: 81/1156 vs. 129/1174 RR (95% CI): 0.53 (0.34–0.81) **SVR and non-SVR compared to no therapy—RR (95% CI):** SVR: RR = 0.15 (0.05–0.45) Non-SVR: RR = 0.57 (0.37–0.85) Number needed to treat to prevent one case of HCC: eight patients.
Simmons 2015 [28]	**Quality of systematic Review** AMSTAR: 8/11 **GRADE Certainty of Evidence** Very low to low	Systematic Review 1990–2014 N = 31 studies: General population: 17 studies; Cirrhotic: nine studies; HIV co-infected: five studies	Adults (>18 years old) chronically infected with HCV of any genotype treated with any antiviral regimen stratified into 3 groups: 1- patients at any disease stage 2- cirrhotic patients 3- HIV/HCV co-infected Total: 33,336 participants General population: 28,398 Cirrhotic: 2604 HIV co-infected: 2358	**Intervention:** PR; IFN; PEG-IFN; IFN-beta **Outcome:** all-cause mortality; pooled adjusted HR (95% CI); pooled estimates for the 5-year mortality	Mortality of achieved SVR vs. non-SVR, aHR (95% CI): General population: 0.50 (0.37–0.67) Cirrhotic group: 0.26 (0.18–0.74) HIV co–infected group: 0.21 (0.10–0.45) Pooled 5-year mortality rates for SVR vs. non–SVR, IR (95% CI): General population: 1.98 (1.00–3.45) vs. 7.75 (5.86–10.98) Cirrhotic group: 4.90 (3.45–7.28) vs. 15.88 (11.44–21.80) Co–infected group: 1.49 (0.50–2.96) vs. 11.44 (6.33–19.3)
Public Health Agency of Canada, Canadian Task Force on Preventative Health Care 2016 [29]	**Quality of systematic Review** AMSTAR: 11/11 **GRADE Certainty of Evidence** Very low to moderate	Systematic Review Up to November 2015 N = Benefits of treatment: 11 studies; Harms of treatment: 7	Treatment-naïve nonpregnant HIV/HBV negative adults Wide range of fibrosis scores +80% noncirrhotic RCTs (n = 7) 6/7 RCTs all patients were Genotype 1 2431 participants ranged from 121 to 499 participants in a study. Recruitment sites included: United States, Australia, Austria, Belgium, Canada, Denmark, France, Germany, New Zealand, Norway, Poland, Russia, Spain, Japan, Italy, Mexico, Puerto Rico, Romania, Ukraine, United Kingdom, Sweden, the Netherlands, Bulgaria, Portugal, Slovakia, China, and the Republic of Korea.	**Intervention:** DAA-based vs. PR regimens. DDA therapies included those that were approved at the time of the study and those anticipated to be approved by February 2016 for all HCV genotypes. **Outcomes:** All-cause mortality; hepatic mortality; hepatic decompensation; hepatocellular carcinoma; need for liver transplantation.	Hepatic mortality: 60 fewer/1000 (95% CI 59–62) Hepatocellular carcinoma: 18 fewer/1000 (17–19) Decompensated cirrhosis: 46 fewer/1000 (46–47) Need for liver transplantation: 4 fewer/1000 (4–6) In cirrhotic individuals DAA–based regimens compared to PR resulted in 30 fewer/1000 people affected by hepatic mortality.
Yehia 2014 [30]	**Quality of systematic Review** AMSTAR: 3/11 Data quality not formally assessed	Systematic Review 2003–2013 N = 10 studies	Only studies from the US that collected data after 2000 were included. Studies of the general population excluded those with only a single study site, exclusively focused on specific populations (e.g., only immigrants, injection drug users, those with HIV/HCV co-infection) **Study subjects for each question** Chronic infection: 15,079 Diagnosed/Aware: 203 Access to Care: 101 HCV RNA confirmed: 8810 Liver biopsy: 180,703 Prescribed HCV treatment: 46,452 Achieved SVR: 18,105	Examined data addressing seven key steps along the HCV care and treatment cascade	**Care/Treatment cascade:** 100% Chronic HCV infected (3,500,000) 50% Diagnosed and aware 43% Access to outpatient care 27% HCV RNA confirmed 17% Underwent liver biopsy 16% Prescribed HCV treatment 9% Achieved SVR

AMSTAR: A MeaSurement Tool to Assess systematic Reviews [22]; aHR: adjusted hazard ratio; CI: confidence interval; CIA: chemiluminescence immunoassay; DAA: direct acting antiviral; EIA: enzyme immunoassay; GRADE: the grading of recommendation assessment, development and evaluation; HCC: hepatocellular carcinoma; HCV: hepatitis C virus; HIV: human immunodeficiency virus; HR: hazard ratio; IFN: interferon; IR: incidence rate; LR: likelihood ratio; NAT: nucleic acid test; OR: odds ratio; PEG-IFN: pegylated interferon; PR: pegylated-interferon-ribavirin; RBV: ribavirin; RCT: randomized controlled trial; RIBA: recombinant immunoblot assay; RNA: ribonucleic acid; RR: risk ratio; SVR: sustained virological response; US: United States; WHO: World Health Organization.

**Table 2:** Included studies on the cost, resource use, and cost-effectiveness of HCV screening and direct acting antiviral therapies.

Study	Quality/Certainty of Economic Evidence	Design/Population	Intervention(s)	Cost-Effectiveness (ICER or INB)	Resource Requirements
**Cost-effectiveness of HCV Screening and DAA therapy**					
Brett-Major 2016 [41]	Certainty of evidence: moderate Allowance was made for uncertainty in the estimates of costs, HCV rates, and ranges were provided. Threshold sensitivity analysis undertaken. PSA not performed. Justification for choice of ranges was provided for all parameters. Cost offsets (and net savings) rather than cost-effectiveness was reported	**Design:** Decision-analytic costing model; results reported in US dollars **Population:** Applicants to US military service HCV prevalence 0.48–0.98/1000	**Three strategies:** 1- Enzyme immunoassay (EIA) screening 2- EIA + nucleic acid testing (NAT) screening 3- no screening Treatment with SOF based regimens	Not applicable (Costing study)	High costs. With no screening, the cost to the Department of Defence of treating the estimated 93 cases of chronic HCV cases from a single year’s accession cohort was $9.3 million [€7,293,134]. Screening with the HCV antibody test followed by the nucleic acid test for confirmation yielded a net annual savings and a $3.1 million dollar [€2,431,044] advantage over not screening.
He 2016 [42]	Certainty of evidence: moderate Allowance was made for uncertainty in the estimates of costs and consequences, and ranges were provided. PSA not performed. Justification for choice of ranges was not provided for all parameters. Cost-effectiveness results were sensitive to the time horizon.	**Design:** Dynamic microsimulation model of transmission/progression of HCV, and cost-effectiveness and budget impact analysis; results reported in US dollars **Population:** Population in US prisons HCV prevalence 25% and 50% undiagnosed	Three strategies: 1- risk-based screening 2- universal opt-out screening 3- no screening Treatment with SOF based regimens	ICER ($/QALY gained): **1 year risk-based vs. no screening:** $19,635 [€15,552] **1 year universal vs. no screening:** $20,571 [€16,293] **5 year universal vs. no screening:** $24,046 [€19,046] **10 year universal vs. no screening:** $29,234 [€23,155]	Low to moderate costs. **Screening cost per 2 million prisoners:** 1year risk-based vs. no screening: +$37M [€29M] 1year universal vs. no screening: +$107M [€84M] 5year universal vs. no screening: +$178M [€140M] 10year universal vs. no screening: +$249M [€197M] **Treatment cost per 2 million prisoners:** No screening: $59,035M [€46,759M] 1year risk-based vs. no screening: +$816M [€646M] 1year universal vs. no screening: +$1480M [€1172M] 5year universal vs. no screening: +$1951M [€1545M] 10year universal vs. no screening: +$2190M [€1734M]
Orkin 2016 [43]	Certainty of evidence: low Allowance was not made for uncertainty in the estimates of costs and consequences. No source for unit prices (costs) was given. PSA not performed. Justification for choice of ranges was not provided for all parameters. Cost-effectiveness results were not reported.	**Design:** Prospective 1 week-long snapshot observational study with assumed costs for testing and treating; results reported in British pounds **Population:** People visiting emergency departments in the UK HCV prevalence 1.84%	**One strategy:** Routine combined HIV, HCV, and HBV testing	Not applicable	Low to moderate costs. Assuming the cost per diagnosis is £7 [€8], the cost per new case detected would be £988 [€1109] for HCV, £1351 [€1517] for HBV. and £2478 [€2783] for HIV.
Rein 2015 [36]	Certainty of evidence: high Allowance was made for uncertainty in the estimates of costs and consequences, and ranges were provided. PSA was performed. Justification for choice of ranges was provided for all parameters. Cost-effectiveness results were sensitive to treatment cost, SVR probability, QALY post SVR, fibrosis rate	**Design:** Monte Carlo simulation model; results presented in US dollars. **Population:** General population aged ≥20, and patients with chronic HCV genotype 1, 2, 3, and 4 in US HCV prevalence rate: varies by birth decade, race, and sex Heavy alcoholics 0.089 HIV+ 0.02	Screening followed by treatment **Five strategies:** 1- PR 2- PI+PR 3- SOF+PR 4- SOF+SIM 5- SOF+RBV	**Genotype ¼—ICER ($/QALY):** PR vs. no treatment: $59,792 [€47,359] PR extensively dominated by PI+PR PI + PR vs. no treatment:$43,530 [€34,478] SOF+PR vs. PI+PR: $47,237 [€37,414] SOF+SIM vs. SOF+PR: $72,169 [€57,162]	Treatment costs: **Genotype 1&4:** PR: $61,224 [€48,493] PI+PR: $78,812 [€62,424] SOF+PR: $99,306 [€78,656] SOF+SIM: $150,360 [€119,094] **Genotype 2:** PR: $30,612 [€24,246] SOF+RBV: $88,158 [€69,826] **Genotype 3:** PR: $30,612 [€24,246] SOF+RBV: $176,316 [€139,653] Other costs: Testing: antibody: $25 [€19] RNA: $59 [€45] Post-diagnostic evaluation: if coordinated with treatment: $832 [€658] if not treated: $869 [€688]
Selvapatt 2015 [44]	Certainty of evidence: moderate Allowance was made for uncertainty in the estimates of costs and consequences, and ranges were provided. PSA not performed. Justification for choice of ranges was not provided for all parameters. Cost-effectiveness results were sensitive to the prevalence of HCV infection among the screened women and the proportion of identified women treated.	**Design:** Markov cohort simulation model; results reported in British pounds **Population:** Pregnant women attending antenatal clinics in the UK HCV prevalence 0.38%	**Two strategies**: 1- screening and treatment (PR) 2- no screening and no treatment Base-case: Treatment with PR based regimen (IFN/RBV) Additional scenarios: 1- IFN/RBV+SOF 2- IFN/RBV → Ø SVR → IFN/RBV+SOF	ICER (£/QALY): **Screening + PR vs. no screening + no treatment:** £2400 [€2745] (**screening + newer direct-acting antiviral regimens vs. no screening + no treatment:** £9139 [€10,455])	Moderate costs. Total costs of screening and confirmation of 44 new diagnoses: £240,641 [€275,299] Cost per newly diagnosed individual: £5469 [€6256]
Wong 2015 [32]	Certainty of evidence: moderate Allowance was made for uncertainty in the estimates of costs and consequences, and ranges were provided. PSA was performed. Justification for choice of ranges was provided for all parameters. Cost-effectiveness results were sensitive to rates of chronic HCV infection, seroprevalence, costs (excluding the cost of antiviral therapy), treatment uptake and quality of life (utilities).	**Design:** Decision-analytic Markov model; results reported in Canadian dollars **Population:** General Canadian population, 2 age groups: 25–64 and 45–64 years old HCV prevalence assumed is 0.5%	**Four strategies:** 1- No screening 2- Screen and treat with PR 3- Screen and treat with: a G1: Interferon free DAA b G2/3: SOF+RBV c G4/5/6: PR 4- Screen and treat with: a G1: SIM+PR b G2/3: SOF+RBV c G4/5/6: PR	ICER (CAN $/QALY) of screening and treatment vs. no screening: **Age 25–64 years:** PR: $38,117 [€25,502] IFN-free DAA (genotype 1), SOF+RBV (genotype 2/3),or PR (genotype 4/5/6): $34,783 [€23,271] PR+RBV (genotype 1), SOF+RBV (genotype 2/3), or PR (genotype 4/5/6): $42,398 [€28,366] **Age 45–64 years:** PR: $34,359 [€22,988] SIM+PR (G1), SOF+RBV (G2/3), or PR (G4/5/6): $44,034 [€29,461] IFN-free DAA (G1), SOF+RBV (G2/3), or PR (G4/5/6): $35,562 [€23,793]	Moderate to high costs: CAN$70,000 [€46,834] $84,000 [€56,201]/person. **Costs of antiviral therapies (CAN $):** SIM-based: 24 weeks: $46,157 [€30,881]; 48 wk: $55,811 [€37,340] SOF-based: 12 wk: $55,000 [€36,798] ABT-based: 48 wk: $19,948 [€13,346]; 24 wk: $9974 [€6673] **Costs of adverse events (weekly):** anemia: $107 [€71], depression: $73 [€48], pruritus: $12 [€8], rash: $12 [€8] **HCV-tests:** anti-HCV: $14 [€9], HCV RNA: $100 [€66] **For age 25–64 years,** No screening: $71,327 [€47,722]; Screen and treat: PR: $71,450 [€47,804] IFN-free DAA (G1), SOF+RBV (G2/3) or PR (G4/5/6): $71,593 [€47,900] SIM+PR (G1), SOF+RBV (G2/3) or PR (G4/5/6): $71,593 [€47,900] **For age 45–64:** No screening: $83,335 [€55,756] Screen and treat: PR: $83,476 [€55,850] IFN-free DAA (G1), SOF+RBV (G2/3) or PR (G4/5/6): $83,672 [€55,981] SIM+PR (G1), SOF+RBV (G2/3) or PR (G4/5/6): $83,673 [€55,982]
**Cost-effectiveness of DAA therapy**					
Chhatwal 2015 [34]	Certainty of evidence: high Allowance was made for uncertainty in the estimates of costs and consequences, and ranges were provided. PSA was performed. Justification for choice of ranges was provided for all parameters. Cost-effectiveness results were most sensitive to quality of life after successful treatment, cost of SOF, drug efficacy	**Design:** Decision-analytic Markov model; results reported in US dollars **Population:** Treatment naïve and treatment-experienced HCV population in US	**Two strategies:** 1- SOF-LDV 2- IFN-based therapy	SOF-LDV vs. IFN-based therapy—ICER ($/QALY): **Treatment naïve patients:** No cirrhosis: $61,517 [€48,725.] Cirrhosis: $20,673 [€16,374] **Treatment experienced patients:** No cirrhosis: $69,707 [€55,212] Cirrhosis: $92,302 [€73,109]	Treating eligible HCV patient would cost an additional $65 billion [€51.5 billion] over a 5 year period **The weekly costs by third-party payer** SOF: $7000 [€5544] LDV: $875 [€693] PEG-IFN: $587 [€464] RBV: $309 [€244] BOC: $1100 [€871] TEL: $4100 [€3247]
Deuffic-Burban 2016 [31] *	Certainty of evidence: moderate Allowance was made for uncertainty in the estimates of costs and consequences, and ranges were provided. PSA not performed. Limited justification for choice of ranges. Cost-effectiveness results were sensitive to the price of new DAAs particularly for treating genotype 1	**Design:** Decision-analytic Markov model; results reported in Euros **Population:** Patients with chronic HCV aged ≥18, aware of their infection, in fibrosis stage F0–F4 or decompensated cirrhosis, treated in France. HCV prevalence	**Three strategies:** 1- TVR/BOC-based triple therapy for genotype 1 and dual therapy with PR for genotypes other than 1 (at F2) 2- SOF/SIM+PR 3- IFN-free DAAs with or without RBV Strategies 2 & 3 evaluated starting treatment at ≥F3, ≥F2 or regardless of fibrosis	**Genotype 1:** IFN-free was a cost-effective vs. IFN-based: ICER: €40,400 to €88,300/QALY QALY/person: 12.59 vs. 12.11 for IFN-based therapy **Genotypes 2 or 3:** IFN-based was the most cost-effective: ICER: €21,300/QALY for genotype 2 ICER: €19,400/QALY for genotype 3 **Genotype 4:** IFN-free regimens was cost-effective: ICER: €23,000 to €58,200/QALY	Moderate to high resource Treating all CHC-screened patients over 5 years would cost: €3.5–7.2 billion **Cost of treatment/week:** SOF: €3417 OBV/PTV-r: €3259 DCV: €2125 SIM: €1750 TEL: €1042 LDV: €417 BOC: €378 DAV: €284 PEG-IFN: €158 RBV: €55 Costs related to adverse events (cost per event): Severe anaemia: €2564 Severe depression: €1619 Severe rash: €2942 Moderate anaemia: €4200
Hagan 2014 [37]	Certainty of evidence: moderate Allowance was made for uncertainty in the estimates of costs and consequences, and ranges were provided. PSA not performed. Justification for choice of ranges was provided for all parameters. Cost-effectiveness results were sensitive to SVR rates	**Design:** Decision-analytic Markov model; results reported in US dollars **Population:** Chronic HCV genotype 1, in 50 years old in US HCV prevalence 1.6%	**Two strategies:** 1- SOF/RBV 2- SOF/SIM	SOF-SIM dominated SOF-RBV: yielded lower costs and more QALYs SOF-SIM: $165,336 [€133,108] and 14.69 QALYs SOF-RBV: $243,586 [€196,106] and 14.45 QALYs	Costs of drugs per course: 24-weeks SOF/RBV: $169,000 12-weeks SOF/SIM: $150,000 Treatment-associated medical care: SOF/RBV: $2100 (1890–2310) [€1690 (€1521–€1859)] SOF/SIM: $1160 (1044–1276) [€933 (€840–€1027)]
Leidner 2015 [40]	Certainty of evidence: moderate Allowance was made for uncertainty in the estimates of costs and consequences, and ranges were provided. PSA not performed. Justification for choice of ranges was provided for all parameters. Cost-effectiveness results were sensitive to post-treatment quality of life (utilities) and treatment costs.	**Design:** Decision-analytic Markov model; results reported in US dollars **Population:** 55-year old patient in US with genotype 1 HCV infection	**Two strategies:** 1- treatment at fibrosis stages F3 and F4 2- treatment strategies at earlier stages of liver disease (fibrosis stages F2, F1, or F0).	ICER ($/QALY): **Patients diagnosed at F0:** treatment at F2 vs. F3: $97,900 [€80,102] treatment at F0 vs. F2: $242,900 [€198,741] **Patients diagnosed at F1:** treatment at F2 vs. F3: $59,500 [€48,683] treatment at F1 vs. F2: $174,100 [€142,449] **Patients diagnosed at F2:** treatment at F2 vs. F3: $37,300 [€30,518] The threshold of treatment costs: for ICER $50,000/QALY: $20,200 [€16,527] for ICER $100,000/QALY: $42,400 [€34,691]	Moderate to high costs. Larger costs for patients with advanced or end stage liver disease, compared to early stage liver disease. Nontreatment and treatment costs: **Patients starting at F0:** treatment at F3: $33,600 [€27,491] treatment at F2: $45,000 [€36,819] treatment at F1: $70,800 [€57,928] treatment at F0: $11,100 [€9082] **Patients starting at F1:** treatment at F3: $59,200 [€48,437] treatment at F2: $77,400 [€63,328] treatment at F1: $113,200 [€92,620] **Patients starting at F2:** treatment at F3: $91,000 [€74,456] treatment at F2: $113,600 [€92,947]
Linas 2015 [39]	Certainty of evidence: high Allowance was made for uncertainty in the estimates of costs and consequences, and ranges were provided. PSA was performed. Justification for choice of ranges was provided for all parameters. Cost-effectiveness results were sensitive to cost of SOF	**Design:** Monte Carlo simulation. Results reported in US dollars **Population:** Chronic HCV genotype 2 or 3 in the US	**Three strategies:** 1- SOF 2- PR 3- No therapy	ICER ($/QALY) **Genotype 2** **No cirrhosis (naïve):** 24 wk PR vs. no therapy: $3000 [€2415] 12 wk SOF-RBV vs. 24 wk PR: $238,000 [€191,609] **No cirrhosis (treatment experienced):** 12 wk SOF-RBV vs. no therapy: $63,700 [€51,283] 16 wk SOF-RBV vs. 12 wk SOF-RBV: $468,000 [€376,777] **Cirrhosis (treatment naïve):** 24 wk PR vs. no therapy: $8700 [€7004] 12 wk SOF-RBV vs. 24 wk PR: $35,500 [€28,580] **Cirrhosis (treatment experienced):** 12 wk SOF-RBV dominated by no therapy SOF-RBV 16 wk vs. 12 wk: $27,300 [€21,978] **Genotype 3** **No cirrhosis (treatment-naïve):** 24 wk PR vs. no treatment: $4800 [€3864] 12 wk SOF-RBV dominated by 24 wk PR 12 wk PR-SOF vs. 24 wk PR: $263,000 [€211,736] 24 wk SOF-RBV vs. 12 wk PR-SOF: $266,000 [€214,151] **No cirrhosis (treatment-experienced):** 12 wk PR-SOF vs. no treatment: $82,000 [€66,016] 12 wk SOF-RBV and 16 wk SOF-RBV both dominated by 12 wk PR-SOF SOF-RBV 24 wk vs. 16 wk: $1,100,000 [€805,080] **Cirrhosis (treatment-naïve):** 24 wk PR vs. no treatment: $13,600 [€10,949] 12 wk SOF-RBV dominated by 12 wk PR 12 wk PR-SOF vs. 24 wk PR: $22,600 [€18,194] 24 wk SOF-RBV vs. 12 wk PR-SOF: $107,000 [€86,143] **Cirrhosis (treatment-experienced):** 12 wk PR-SOF vs. no treatment: $22,300 [€17,953] 12 wk, 16 wk and 24 wk SOF-RBV all dominated by 12 wk PR-SO	**Total HCV therapy costs per number of weeks (base case value and range from sensitivity analysis):** 24 wk PR: $25,300 (12,800–37,800) [€20,368 (10,305–30,432)] 12 wk SOF–RBV: $91,500 (2000–97,500) [€73,664 (1610–78,495)] 16 wk SOF–RBV: $121,900 (30,000–129,900) [€98,139 (24,152–104,579)] 24 wk SOF–RBV: $182,900 (4900–194,900) [€147,249 (3944–156,910)] 12 wk PR-SOF: $9000 (3000–$105,000) [€7245 (2415–84,533)]
Najafzadeh 2015 [33]	Certainty of evidence: high Allowance was made for uncertainty in the estimates of costs and consequences, and ranges were provided. PSA was performed. Justification for choice of ranges was provided for all parameters. Cost-effectiveness results were sensitive to treatment cost	**Design:** Discrete event simulation **Population:** Treatment-naive patients infected with chronic HCV genotype 1, 2, or 3 in the US.	**Five strategies** (genotype 1) 1- BOC+PR 2- SOF+PR 3- SOF+SIM 4- SOF+DCV 5- SOF+LDV **4 strategies** (genotype 2/3): 1- PR 2- SOF+RBV 3- SOF+DCV 4- SOF+LDV+ RBV (genotype 3 only)	ICER ($/QALY): **Genotype 1** BOC+PR: reference SOF+PR: $21,528 [€17,051] SOF+SIM: $71,445 [€56,589] SOF+DCV: $63,355 [€50,181] SOF+LDV: $12,825 [€10,158] **Genotype 2:** PR: reference SOF+RBV: $110,168 [€87,260] SOF+DCV: $691,574 [€48,770] **Genotype 3:** PR: reference SOF+RBV: dominated by PR SOF+DCV: $396,229 [€313,839] SOF+LDV+RBV: $73,236 [€58,007]	Drug costs: **Genotype 1:** BOC+PR: $100,926 [€79,940] SOF+PR: $120,648 [€95,561] SOF+SIM: $171,023 [€135,461] SOF+DCV: $169,747 [€134,450] SOF+LDV: $115,358 [€91,371] **Genotype 2:** PR: $54,005 [€42,775] SOF+RBV: $109,958 [€87,093] SOF+DCV: $316,845 [€250,962] **Genotype 3:** PR: $58,323 [€46,195] SOF+RBV: $207,872 [€164,648] SOF+DCV: $317,830 [€251,742] SOF+LDV+RBV: $120,464 [€95,415]
Saab 2014 [38]	Certainty of evidence: moderate Allowance was made for uncertainty in the estimates of costs and consequences, and ranges were provided. PSA was performed. Justification for choice of ranges was not provided for all parameters. Cost-effectiveness results were most sensitive to cirrhosis prevalence and fibrosis rate, recurrence rates in patients achieving SVR.	**Design:** Decision-analytic Markov model; results reported in US dollars **Population:** Patients with chronic HCV genotype 1 in US	**Five Strategies:** 1- SOF+PR 2- PR 3- BOC+PR 4- TEL+PR 5- SIM+PR	ICER ($/QALY): **Treatment naïve (without cirrhosis)** SOF+PR compared with PR: ≤$29,271 [€23,565] No treatment: $2071 [€1667] SOF+PR dominated BOC+PR, TEL+PR and SIM+PR **Treatment naïve (with cirrhosis)** SOF+PR compared with PR: ≤$16,939 [€13,637] BOC+PR: $8450 [€6802] SIM+PR: $1899 [€1528] No treatment: $17,299 [€13,927] SOF+PR dominated TEL+PR **Treatment experienced (all patients)** SOF+PR compared with PR: ≤$4290 [€3453] No treatment: $16,617 [€13,378] SOF+PR dominated BOC+PR, TEL+PR and SIM+PR	Total lifetime costs: **Treatment-naïve without cirrhosis:** SOF+PR: $116,715 [€93,964] PR: ≤$95,333 [€76,750] BOC+PR: $124,229 [€100,014] TEL+ PR: $128,879 [€103,757] SIM+PR: $120,318 [€96,865] No treatment: $112,093 [€90,243] **Treatment-naïve with cirrhosis:** SOF+PR: $209,923 [€169,004] PR: ≤$172,814 [€139,129] BOC+PR: $199,192 [€160,365] TEL+ PR: $211,996 [€170,673] SIM+PR: $207,758 [€167,261] No treatment: $140,210 [€112,880] **Treatment-experienced all patients:** SOF+PR: $148,812 [€119,805] PR: ≤$145,009 [€116,743] BOC+PR: $165,983 [€133,629] TEL+ PR: $165,428 [€133,182] SIM+PR: $168,251 [€135,455] No treatment: $115,911 [€93,317]
Younossi 2015 [35]	Certainty of evidence: moderate Allowance was made for uncertainty in the estimates of costs and consequences, and ranges were provided. PSA was performed. Justification for choice of ranges was not provided for patient distribution, regimen efficacy, costs, or utilities. Cost-effectiveness results were robust across the limited ranges tested.	**Design:** Decision-analytic Markov model; results reported in US dollars **Population:** Patients with chronic HCV genotype 1 in US.	**Six strategies:** 1- LDV/SOF 2- SOF+PR 3- SIM+PR 4- SOF+SIM 5- SOF+RBV 6- BOC+PR	LDV/SOF (ICER): **Treatment-naïve patients:** dominant over no treatment dominant over SOF+PR (12/24 weeks) less expensive and less effective than SOF+SIM dominant over SOF+RBV dominant over BOC+PR Results similar for patients with and without cirrhosis; and for treatment experienced patients with PR or Protease inhibitor (PI) + RBV	Drug costs/pack: BOC: $6687 [€5296] LDV/SOF: $31,500 [€24,950] PEG-IFN: $3310 [€2621] SIM: $22,120 [€17,520] SOF: $28,000 [€22,177] RBV: $1153 [€913]; Generic: $238 [€188] **Total lifetime costs by strategy (treatment naïve):** No treatment: $141,856 [€112,359] LDV/SOF: $90,127 [€71,386] SOF+PR: $119,846 [€94,925] SIM +PR: $128,793 [€102,012] SOF+SIM: $191,631 [€151,784] SOF+RBV: $229,200 [€181,541] BOC+PR: $127,759 [€101,193]

ABT: a protease inhibitor; BOC: boceprevir; CAD: Canadian dollar; DAA: direct acting antiviral; DAV: dasabuvir; DCV: daclatasvir; F: fibrosis stage; G: genotype; HBV: hepatitis B virus; HCV: hepatitis C virus; HIV: human immunodeficiency virus; ICER: incremental cost-effectiveness ratio; IFN: interferon; INB: incremental net benefit LDV: ledipasvir; OBV: ombitasvir; PI: protease inhibitor; PEG-IFN: pegylated interferon; PR: pegylated-interferon-ribavirin; PSA: probabilistic sensitivity analysis; PTV-r: paritaprevir-ritonavir; QALY: quality-adjusted life years; RBV: ribavirin; RNA: ribonucleic acid; SIM: simeprevir; SOF: sofosbuvir; SVR: sustained virological response; TEL/TVR: telaprevir; US: United States. * Costs were expressed in 2015 Euro in the original publication.

## Supplementary Materials

The following are available online at http://www.mdpi.com/1660-4601/15/9/2013/s1, Figure S1: Analytic Framework for HCV Screening in Migrants; Table S1. Effectiveness and Cost-effectiveness Search Strategy; Tables S2–S5. Study profile GRADE; Table S6. Chronic HCV burden in migrants: the 10 migrant groups from intermediate/high HCV prevalence countries with the highest number of HCV cases in host EU/EEA countries.
