# Supplementary material for: The Effectiveness and Cost-Effectiveness of Hepatitis C Screening for Migrants in the EU/EEA: A Systematic Review
Source: Int J Environ Res Public Health. 2018 Sep 14;15(9):2013. doi: 10.3390/ijerph15092013 (PMC6164358; doi:10.3390/ijerph15092013)
Supplement: Supplementary file 1 [file ijerph-15-02013-s001.pdf]

International Journal of
Environmental Research and Public Health

MDPI

APPENDIX: Effectiveness and cost effectiveness of hepatitis C screening for migrants in the EU/EEA

Contents

APPENDIX 1. Figure S1: Analytic Framework for HCV Screening in Migrants 2

APPENDIX 2. Table S1: Effectiveness and Cost-effectiveness Search Strategy 3

APPENDIX 3. Table S2–S5: Study profile GRADE 5

APPENDIX 4. Table S6: Chronic HCV burden in migrants: The 10 migrant groups from intermediate/high HCV prevalence countries with the highest number of HCV cases in host EU/EEA countries 10

APPENDIX 1. Figure S1: Analytic Framework for HCV Screening in Migrants

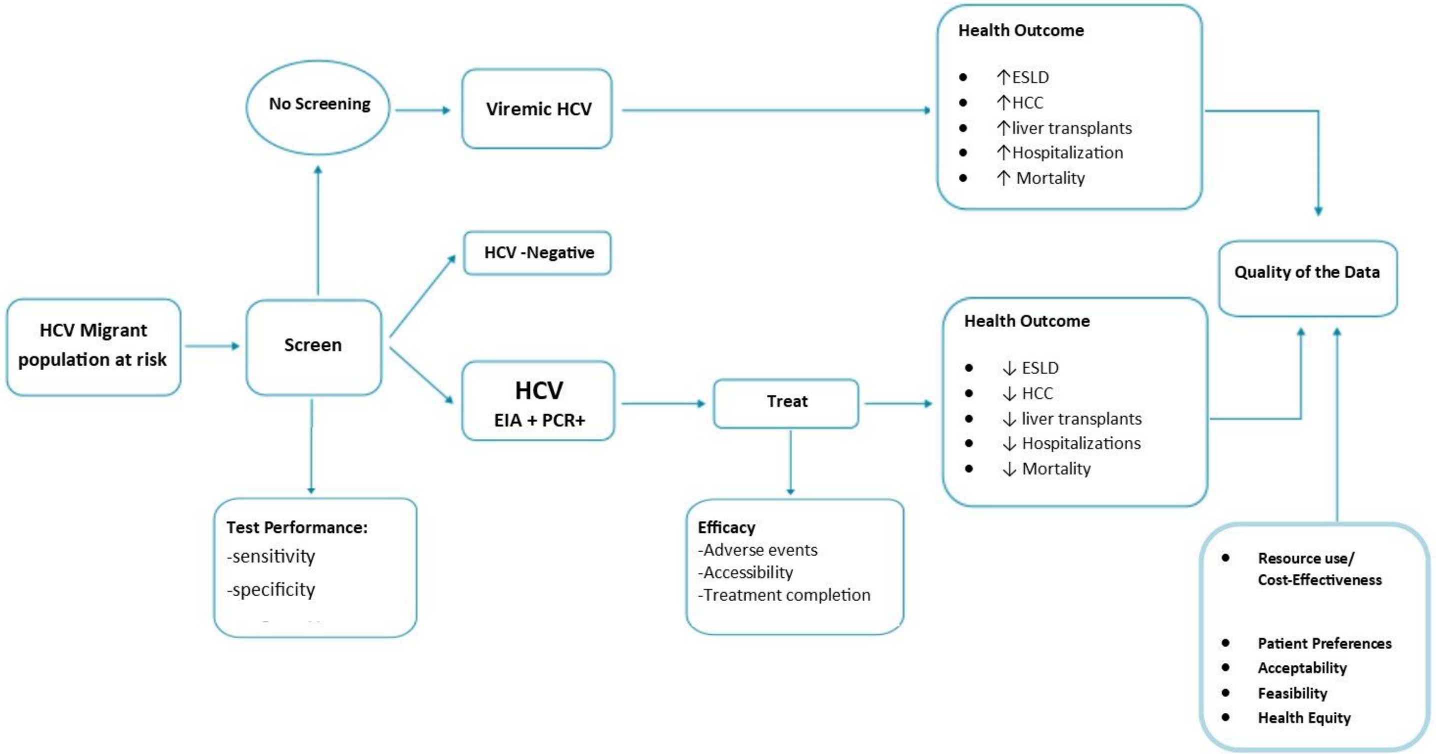

Figure S1. Analytic Framework for HCV Screening in Migrants. EIA: enzyme immunoassay; ESLD: end-stage liver disease; HCC: hepatocellular carcinoma; HCV: hepatitis C virus; PCR: polymerase chain reaction.

APPENDIX 2. Table S1: Effectiveness and Cost-effectiveness Search Strategy

Table S1. Effectiveness and Cost-effectiveness Search Strategy.

Database: Ovid MEDLINE(R) 1946 to Present with Daily Update
Search Date: 12 May 2016

1	exp Hepatitis C/ (52799)
2	(CHC or HCV or HepC).mp. (43097)
3	((hep or hepatitis) adj3 C).mp. (70370)
4	or/1-3 (74482)
5	exp Mass Screening/ (108318)
6	(screened or screening? or tested or testing or tests).tw. (1693692)
7	Early Diagnosis/ (19242)
8	((case? or early) adj2 (detected or detection? or diagnos$ or discover$)).tw. (150411)
9	exp Population Surveillance/ (56471)
10	(disease? adj2 surveillance).tw. (4099)
11	Contact Tracing/ (3546)
12	contact tracing.tw. (1157)
13	or/5-12 (1898063)
14	meta analysis.mp,pt. (92974)
15	review.pt. (2047386)
16	search$.tw. (257066)
17	guideline.pt. (15756)
18	guideline/ (15756)
19	guidelines as topic/ (33974)
20	practice guideline.pt. (21165)
21	practice guideline/ (21165)
22	practice guidelines as topic/ (91485)
23	(CPG or CPGs or guidance or guideline? or recommend$ or standard?).ti. (144070)
24	exp clinical pathway/ (5254)
25	exp clinical protocol/ (138943)
26	((care or clinical) adj2 pathway?).tw. (4952)
27	or/14-26 (2545831)
28	4 and 13 and 27 (2387)
29	animals/ not (humans/ and animals/) (4208789)
30	28 not 29 (2378)
31	30 and (2010$ or 2011$ or 2012$ or 2013$ or 2014$ or 2015$ or 2016$).ed. (810)
32	remove duplicates from 31 [reviews and guidelines] (788)
33	exp "costs and cost analysis"/ (197506)
34	cost$.mp. (457033)
35	cost effective$.tw. (80835)
36	cost benefit analys$.mp. (67070)
37	health care costs.mp. (36863)
38	or/33-37 (466345)
39	4 and 13 and 38 (810)
40	animals/ not (humans/ and animals/) (4208789)
41	39 not 40 (808)

42 41 and (2010$ or 2011$ or 2012$ or 2013$ or 2014$ or 2015$ or 2016$).ed. (327)
43 remove duplicates from 42 [costing] (313)
****

APPENDIX 3. Table S2–S5: Study profile GRADE

GRADE Table S2: Diagnostic accuracy of point-of-care tests for hepatitis C virus infection: a systematic review and meta-analysis.

Quality assessment							Outcome	Certainty of evidence (GRADE)	Importance
№ of studies	Study design	Risk of bias	Inconsistency	Indirectness	Imprecision	Other considerations			
Sensitivity of point of care testing vs laboratory testing									
30	observational studies [a]	serious [b]	serious [c]	not serious	not serious	none	Sensitivity = 97.5% (95% CI: 95.9–98.4)	⊕◯◯◯ VERY LOW	CRITICAL
Specificity of point of care testing vs laboratory testing									
30	observational studies [a]	serious [b]	serious [c]	not serious	not serious	none	Specificity= 99.6% (95%CI: 99.3–99.8)	⊕◯◯◯ VERY LOW	CRITICAL
Positive likelihood ratio of point of care testing vs laboratory testing									
30	observational studies [a]	serious [b]	serious [c]	not serious	not serious	none	Positive likelihood ratio= 80.2 (95% CI: 55.4–116.1)	⊕◯◯◯ VERY LOW	CRITICAL
Negative likelihood ratio of point of care testing vs laboratory testing									
30	observational studies [a]	serious [b]	serious [c]	not serious	not serious	none	Negative likelihood ratio= 0.03 (95% CI: 0.02–0.04)	⊕◯◯◯ VERY LOW	CRITICAL

Khuroo et al *PLoS ONE.* 2015;10:e0121450.; **CI:** Confidence interval; Explanations: a. 10 cross sectional, 20 case control; b. Many studies had patient selection bias and lack of blinding. Many studies scored poorly on quality scales.; c. Heterogeneity greater than 85%.

GRADE Table S3. Antiviral therapy for prevention of hepatocellular carcinoma in chronic hepatitis C: systematic review and meta-analysis of randomized controlled trials.

Quality assessment							№ of patients		Effect		Certainty of evidence (GRADE)	Importance
№ of studies	Study design	Risk of bias	Inconsistency	Indirectness	Imprecision	Other considerations	Antiviral therapy	Placebo or no intervention	Relative (95% CI)	Absolute (95% CI)		
Hepatocellular carcinoma in those who took therapy												
8 [a]	randomised trials [a]	serious [b]	not serious	not serious	not serious	none	81/1156 (7.0%)	129/1174 (11.0%)	RR 0.53 (0.34 to 0.81)	52 fewer per 1,000 (from 21 fewer to 73 fewer)	⊕⊕⊕◯ MODERATE	CRITICAL
Hepatocellular carcinoma in those who achieved SVR												
3	randomised trials	serious [b]	not serious	not serious	not serious	none	Not available [c]	Not available [c]	RR 0.15 (0.05 to 0.45)	Not available [c]	⊕⊕⊕◯ MODERATE	CRITICAL
Hepatocellular carcinoma in those who did not achieve SVR												
5	randomised trials	serious	not serious	not serious	not serious	none	Not available [b]	Not available [c]	RR 0.57 (0.37 to 0.85)	Not available [c]	⊕⊕⊕◯ MODERATE	CRITICAL

Kimer et al. BMJ Open 2012;2:e001313. doi:10.1136/bmjopen-2012-001313; **CI:** Confidence interval; **RR:** Risk ratio; Explanations: a. Study included 8 RCTS and 6 cohort studies. However, only higher quality RCT evidence is reported and is supported by cohort stuides findings; b. Downgraded as none of the included trials were blinded and lack of trial registration; c. Data not available in systematic review; only relative risk provided.

GRADE Table S4. Long-Term Treatment Outcomes of Patients Infected With Hepatitis C Virus: A Systematic Review and Meta-analysis of the Survival Benefit of Achieving a Sustained Virological Response.

Quality assessment							№ of patients		Effect		Certainty of evidence (GRADE)	Importance
**№ of studies**	**Study design**	**Risk of bias**	**Inconsistency**	**Indirectness**	**Imprecision**	**Other considerations**	**[intervention]**	**[comparison]**	**Relative (95% CI)**	**Absolute (95% CI)**		
Mortality rates for General cohort (achieving SVR vs not achieving SVR)												
17	observational studies	not serious [a]	serious [b]	not serious	not serious	none	502/12140 (4.1%)	708/16258 (4.4%)	**HR 0.50** (0.37 to 0.67)	**22 fewer per 1,000** (from 14 fewer to 27 fewer)	⨁◯◯◯ VERY LOW	CRITICAL
Mortality rates for Cirrhotic Cohort (achieving SVR vs not achieving SVR)												
9	observational studies	not serious [a]	not serious	not serious	not serious	none	45/778 (5.8%)	404/2108 (19.2%)	**HR 0.26** (0.18 to 0.74)	**138 fewer per 1,000** (from 46 fewer to 154 fewer)	⨁⨁◯◯ LOW	CRITICAL
Mortality rates for Coinfected Cohort (achieving SVR vs not achieving SVR)												
5	observational studies	not serious [a]	not serious	not serious	serious	none	11/857 (1.3%)	161/1501 (10.7%)	**HR 0.21** (0.10 to 0.45)	**84 fewer per 1,000** (from 57 fewer to 96 fewer)	⨁◯◯◯ VERY LOW	CRITICAL

Simmons et al *Clin Infect Dis.* 2015;61(5):730-740 ; **CI:** Confidence interval; **HR:** Hazard Ratio; Explanations: a. 68.2% of domains of all studies showed a low risk of bias based on Quality in Prognosis Studies (QUIPS) tool; b. Heterogeneity higher in this comparison, but decreased with subgroup analysis of non-treatment control groups and treatment control groups.

GRADE Table S5. Efficacy of DAA-based treatment compared to PR (alone) for HCV treatment.

Quality assessment							№ of patients		Effect		Certainty of evidence (GRADE)	Importance
№ of studies	Study design	Risk of bias	Inconsistency	Indirectness	Imprecision	Other considerations	DAA-based treatment	PR (alone)	Relative (95% CI)	Absolute (95% CI)		
Hepatic Mortality												
1	randomized trials	not serious	serious [1]	not serious	not serious	publication bias strongly suspected strong association [2]	29756/600000 (5.0%)	10990/100000 (11.0%)	**RR 0.45** (0.44 to 0.46)	**60 fewer per 1,000** (from 59 fewer to 62 fewer)	⊕⊕⊕◯ MODERATE	CRITICAL
All-cause mortality												
5	randomized trials	serious [3,4]	not serious	serious [5]	serious [6]	publication bias strongly suspected [2]	2/1206 (0.2%)	0/644 (0.0%)	**RR 2.14** (0.23 to 20.01)	**0 fewer per 1,000** (from 0 fewer to 0 fewer)	⊕◯◯◯ VERY LOW	CRITICAL
Hepatocellular Carcinoma												
1	randomized trials	serious [3,4,7,8]	serious [1]	serious [5]	not serious	publication bias strongly suspected strong association [2]	18456/600000 (3.1%)	4890/100000 (4.9%)	**RR 0.63** (0.61 to 0.65)	**18 fewer per 1,000** (from 17 fewer to 19 fewer)	⊕◯◯◯ VERY LOW	CRITICAL
Sustained Virological Response at 12 weeks (SVR 12)												
7	randomized trials	not serious	serious [9]	serious [5]	not serious	publication bias strongly suspected very strong association [2]	1310/1606 (81.6%)	512/822 (62.3%)	**RR 1.29** (1.22 to 1.37)	**181 more per 1,000** (from 137 more to 230 more)	⊕⊕⊕◯ MODERATE	IMPORTANT
Sustained Virological Response at 24 weeks (SVR 24)												

7	randomized trials	not serious	serious [9]	serious [5]	not serious	publication bias strongly suspected very strong association [2]	1302/1606 (81.1%)	503/822 (61.2%)	**RR 1.31** (1.23 to 1.39)	**190 more per 1,000** (from 141 more to 239 more)	⨁⨁⨁◯ MODERATE	IMPORTANT
Sustained Virological Response at 72 weeks (SVR 72)												
1	randomized trials	not serious	serious [10]	serious [5]	not serious	publication bias strongly suspected very strong association [2]	923/1134 (81.4%)	295/493 (59.8%)	**RR 1.36** (1.26 to 1.47)	**215 more per 1,000** (from 156 more to 281 more)	⨁⨁⨁◯ MODERATE	IMPORTANT
Need for transplant												
1	randomized trials	serious [3,8,9]	serious [1]	serious [5]	not serious	publication bias strongly suspected strong association [2]	18456/600000 (3.1%)	4890/100000 (4.9%)	**RR 0.39** (0.35 to 0.42)	**30 fewer per 1,000** (from 28 fewer to 32 fewer)	⨁◯◯◯ VERY LOW	IMPORTANT

Public Health Agency if Canada (PHAC). Treatment of Hepatitis C Virus: a systematic Review and Meta-Analysis.2016; **CI:** Confidence interval; **RR**: Risk ratio.

Reasons for downgrading and/or upgrading the quality of evidence

1. Heterogeneity was not provided in the meta-analysis
2. Funnel plot asymmetry was not provided to assess the publication bias. Because less than 10 studies were included as included- the results may have been impacted by publication bias
3. high risk of bias for performance bias
4. high risk of bias for detection bias
5. The population in this review was treatment-naïve, without HIV or hepatitis B co-infection, without prior liver transplantation, and the majority (over 80%) were non-cirrhotic or did not show evidence of cirrhosis or liver damage
6. Wide confidence intervals
7. High risk of bias for allocation concealment
8. High risk of bias for random allocation
9. High heterogeneity (I-squared=81%)
10. High heterogeneity (I-squared=79%)

Interpreting the Evidence Profile:

- Seven outcomes were included for treatment efficacy of DAA compared to PR (3 outcomes were critical; 3 outcomes were important; 1 outcome was not important)
- Example of assessing the certainty of evidence (hepatic mortality):

- The certainty was downgraded due to inconsistency (heterogeneity cannot be assessed) and publication bias.
- The certainty was upgraded due to the statistically significant large effect [RR 0.45 (95% CI 0.44, 0.46]

- Moderate certainty of evidence on HCV treatment outcomes : hepatic mortality, SVR 12, SVR 24, SVR 72
- Very low certainty of evidence on HCV treatment outcomes: All-cause mortality, HCC, need for transplant

Interpreting Relative & Absolute Values (e.g. hepatic mortality) from the Evidence Profile:

- Relative Risk: [RR 0.45 (95% CI 0.44, 0.46]- the DAA groups showed a relative risk reduction of 55% in hepatic mortality.
- Absolute risk: The absolute reduction in hepatic mortality was 60 fewer per 1,000 (range: 59 to 62) with DAA treatment compared to PR

APPENDIX 4. Table S6: Chronic HCV burden in migrants: The 10 migrant groups from intermediate/high HCV prevalence countries with the highest number of HCV cases in host EU/EEA countries

Table S6. Chronic HCV burden in migrants: The 10 migrant groups from intermediate/high HCV prevalence countries with the highest number of HCV cases in host EU/EEA countries.

Member state	Top migrant groups with HCV by country of origin accounting for ≥70% of HCV cases in migrants	Number (proportion of all migrant HCV cases)
Austria	Romania, Bosnia and Herzegovina, Egypt, Serbia, Turkey, Italy, Russia, Poland, Nigeria, Croatia	9073 (77%)
Belgium	Italy, DR Congo, Morocco, Former Soviet Union, Cameron, Romania, Turkey, Poland, Former Yugoslavia, Spain	13,664 (73%)
Bulgaria	Russia, Ukraine, Romania, Greece, Uzbekistan, Armenia, Moldova, Azerbaijan, Turkey, Syria	1121 (88%)
Croatia	Bosnia and Herzegovina, Serbia, Kosovo, Slovenia, FYR Macedonia, Italy, Montenegro, Russian Federation, Egypt, Switzerland	4795 (99%)
Cyprus	Georgia, Romania, Egypt, Russia, Greece, Bulgaria, Ukraine, Syria, Pakistan, Sri Lanka	2146 (78%)
Czech Republic	Ukraine, Russia, Slovakia, Vietnam, Mongolia, Uzbekistan, Poland, Moldova, Kazakhstan, Romania	5273 (88%)
Denmark	Iraq, Pakistan, Romania, Lebanon, Turkey, Poland, Thailand, Italy, Bosnia and Herzegovina, Lithuania	2517 (65%)
Estonia	Russia, Ukraine, Belarus, Kazakhstan, Uzbekistan, Georgia, Latvia, Lithuania, Azerbaijan, Armenia	5005 (98%)
Finland	Former Soviet Union, Estonia, Russia, Iraq, Thailand, Nigeria, Egypt, China, Former Yugoslavia, Italy	2766 (82%)
France	Algeria, Italy, Morocco, Portugal, Cameron, Senegal, Tunisia, Spain, Egypt, Ivory Coast	63,559 (72%)
Germany	Russia, Poland, Kazakhstan, Italy, Romania, Turkey, Ukraine, Uzbekistan, Greece, Iraq	106,365 (83%)
Greece	Albania, Georgia, Egypt, Russia, Pakistan, Romania, Armenia, Ukraine, Bulgaria, Syria	12,304 (95%)
Hungary	Romania, Ukraine, Former Soviet Union, Serbia, Slovakia, China, Russia, Italy, Egypt, Nigeria	6125 (93%)
Iceland	Poland, Lithuania, Thailand, United States, Latvia, Russia, Italy, Ukraine, Romania, Portugal	158 (77%)
Ireland	Nigeria, Poland, Lithuania, Romania, Pakistan, Latvia, Italy, Egypt, Russia, United States	4113 (75%)
Italy	Romania, Egypt, Albania, Ukraine, Morocco, Moldova, Nigeria, Senegal, Pakistan, Russia	61,134 (78%)
Latvia	Russia, Ukraine, Belarus, Lithuania, Uzbekistan, Georgia, Estonia, Azerbaijan, Moldova	6420 (98%)
Liechtenstein	Switzerland, Italy, Portugal, Turkey, Spain, Bosnia and Herzegovina, Kosovo, Brazil Egypt, Russia	159 (92%)
Lithuania	Russia, Belarus, Ukraine, Kazakhstan, Latvia, Uzbekistan, Georgia, Armenia, Azerbaijan, Estonia	2693 (96%)
Luxembourg	Portugal, Italy, Cape Verde, Romania, Cameroon, Russia, Spain, Montenegro, Bosnia and Herzegovina, Angola	1446 (86%)

Malta	Australia, Egypt, Italy, Russian Federation, Nigeria, Canada, Romania, United States, Somalia, Ukraine	230 (78%)
Netherlands	Morocco, Turkey, Egypt, Former Soviet Union, Iraq, Italy, Poland, Ghana, China, Former Yugoslavia	8902 (67%)
Norway	Pakistan, Poland, Lithuania, Iraq, Russia, Thailand, Romania, Somali, United States, Latvia	3359 (69.6%)

European Centre for Disease Prevention and Control. Epidemiological assessment of hepatitis B and C among migrants in the EU/EEA. Stockhlom: ECDC; 2016 2016.
